# (How) Can Clinical Psychology Contribute to Increasing Vaccination Rates in Europe?

**DOI:** 10.32872/cpe.7525

**Published:** 2021-09-30

**Authors:** Tania M. Lincoln, Winfried Rief

**Affiliations:** 1 Clinical Psychology and Psychotherapy, Institute of Psychology, University of Hamburg, Hamburg, Germany; 2 Division of Clinical Psychology and Psychotherapy, Department of Psychology, Philipps-University of Marburg, Marburg, Germany

The speed in which several vaccines for COVID-19 were developed, approved and rolled out in Europe is amazing. Unfortunately, though, what started out as a story of success is presently being spoilt by the high rate of vaccine hesitancy and refusal. In those European countries that have already offered a vaccine to all adults the rate of vaccine uptake is levelling off well below 70%. While this seemed sufficient at the beginning of the pandemic, the newer virus variants with higher infectiousness require much higher participation rates for vaccination campaigns, and experts now estimate the necessary threshold to be about 90%.

To better understand vaccine hesitancy, numerous researchers have diligently been studying its putative predictors. Sociodemographic variables they found to be consistently associated with hesitancy or refusal were younger age, female gender, lower income, lower education, unemployment, and migrant status (Freeman et al., 2020; Neumann-Böhme et al., 2020; Sallam, 2021; Wake, 2021). Also, people with more extreme political views (Peretti-Watel et al., 2020), higher social media consumption (Allington et al., 2021; Ebrahimi et al., 2021; Murphy et al., 2021), and more frequent use of messenger services and online video platforms (Holzmann-Littig et al., 2021) seem to be less likely to accept a COVID-19 vaccine.

In general, the decision to participate mainly depends on three factors: “benefit” (What kind of benefit do I expect if I participate?), “harm” (i.e. Are these vaccines producing negative effects on my body?), and feasibility (How difficult is it to participate?). In regard to harm expectations, research has identified attitudes indicative of a general mistrust of the government and its institutions (Ebrahimi et al., 2021; Freeman et al., 2020; Lincoln et al., 2021; Murphy et al., 2021), conspiracy beliefs (Allington et al., 2021; Freeman et al., 2020; Murphy et al., 2021), and specific concerns related to vaccine safety and efficacy (Freeman et al., 2020; Neumann-Böhme et al., 2020) to predict vaccine willingness. Related to benefit expectations, it has been shown that the lower people perceive the risk of getting COVID-19, the less willing they are to get vaccinated (Allington et al., 2021; Bono et al., 2021; Ebrahimi et al., 2021).

Interventions to combat vaccine hesitancy have included increasing the incentives (e.g. paying people for vaccination or reducing the options of social participation for refusers), using role models, combatting misinformation in social media, providing patient choice, and providing low-threshold vaccination in socially deprived areas. Although some of these interventions align with the known socio-demographic predictors, surprisingly, none of them seem to focus directly at changing the beliefs driving vaccine hesitancy or refusal. Is this, perhaps, because changing peoples’ beliefs is seen as particularly difficult? Maybe. But not for clinical psychologists! Changing beliefs to motivate adaptive behavior is what we do every day. We do not intend to imply that vaccine hesitancy is indicative of a mental health problem. We do think, however, that the beliefs driving vaccine hesitancy are maladaptive, both from an empirical perspective, as they are not well backed up by evidence, and from a functional perspective, as they are putting people at risk. Classifying these attitudes as maladaptive provides us with a unique opportunity to bring in our expertise to the aim of finally moving out of this pandemic. To make this a bit more concrete, what do you think about the following five-step basic cognitive-behavioral intervention for dealing with vaccine hesitant fellow citizens?

1. **Specifying the problem in order to set a realistic aim**
  According to the German COSMO study (COSMO-Konsortium, 2021), only about half of those not yet vaccinated report to be definite refusers, the other half are either merely unsure or even basically willing. A clear understanding of where someone stands is key to setting a realistic goal and finding the most appropriate intervention. Is it a problem of attitude (e.g. not wanting to get a vaccine) or of behavior (being willing in principle, but not having put this willingness into practice yet)? And if it is a problem of attitude, how pronounced is it? Is someone an absolute refuser and is actively spreading misinformation? In this case, a realistic next step could be to sow some seeds of doubt. Or has someone merely got a couple of specific concerns that are preventing him or her from getting a vaccine? In this case, the aim could be to overcome these doubts and develop a principle vaccination willingness. Finally, for those who are already willing in principle, the aim should be to overcome the practical barriers and actually get the vaccine. Prochaska and Di Clemente’s (1986) motivational model for change provides a helpful framework for defining what the problem is, where the patient is, and what would be a reasonable next step in this case.
2. **Assessing the reasons and delineating the appropriate intervention**
  If the problem is one of attitude, the main concerns need to be explored. Are they related to a profound and generalized mistrust of the government and its institutions or even to conspiracy beliefs or does someone have very specific vaccine safety-concerns? Are we dealing with a fear of side- or long-term effects or with phobic concerns related to the prick of the needle? Is someone clearly underestimating the risk of the pandemic or have they perhaps not understood the differences in probabilities between developing serious side effects, versus getting infected with serious consequences?
  In addition, we need to understand how these attitudes are maintained even if contradicting information is provided. Do people use “cognitive immunization” (Rief & Joormann, 2019) strategies to block any effects of corrective information? In this case, it will not be sufficient to provide new information, but also to challenge or circumvent these cognitive immunization strategies.
  If the problem is one of behaviour, we need to find out what the specific barriers are (Is it, for example, a lack of time? A lack of knowledge about where to go? Or is someone worried about not having the documents that may be required?). Once we have understood the reasons, it will be clearer which type of intervention is the most promising.
3. **Building rapport to motivate behaviour change**
  As any therapist will know, simply telling someone that their beliefs are wrong or unfounded is unlikely to be helpful. It tends to motivate people to defend their beliefs, or even to leave the conversation, never to come back. People have reasons for their beliefs and, in any case, no one can be 100% certain of what is right and wrong. Staying open minded oneself and also expressing authentic understanding for the other persons’ beliefs will create an open space in which a change of perspective is more likely to occur. Even in the case of extreme mistrust or conspiracy beliefs, which may be more difficult to empathize with, it can help to express understanding for the frustration with political decisions and the restrictions.
4. **Challenging beliefs**
  If we are dealing with maladaptive beliefs, we can now follow the basic pattern of cognitive therapy. We begin by narrowing down the beliefs to one core statement. We then assess how certain the person is of the truth of this belief (maybe using a scale from 1 to 100 percent). We can now move on to the empirical dispute by creating a list of evidence for and against the belief. Mind to use guided discovery and let the other person come up with the pros and cons herself. But if you can’t stop yourself from adding to the list, then be sure to add to both sides of evidence. You might also want to discuss the quality of each piece of evidence to help the other person decide which weight to give it. For example, while severe adverse effects are indeed a matter of concern, mild side effects are generally fleeting in nature and easy to cope with.
  Depending on the type of belief, it may also make sense to use functional dispute. This is done by looking at the pros and cons of sticking with the belief versus changing it. Knowing your stats will come in helpful here as this process may well involve weighing up the risk of severe side effects with the risk of a serious COVID-19 infection (Rief, 2021).
  Established and powerful “cognitive immunization” strategies, need to be addressed specifically. These strategies can be categorized into two types: devaluating the source of information (e.g. you cannot believe those people who are in favor of vaccination anyway), and devaluating the content of expectation-violating information (e.g. information violating my beliefs is only based on accidental events). They are also evident on a behavioral level by avoidance of any conflicting information and continuing to live in an information bubble. Again, we need to validate cognitive immunization strategies, because they provide stability in our lives, but they are also the basis of continuous misjudgments, and therefore, searching for alternative information channels is crucial to prevent tunnel perspectives.
  This process can be concluded by re-assessing the conviction in the belief to see where you stand now. Don’t worry if it hasn’t changed much, after all its not about winning an argument, but about helping someone to take different aspects into account. According to Prochaska and Di Clemente’s (1986) model, it’s just about moving to the next level of change, not about completely convincing a person in one blow.
5. **Overcoming behavioral barriers**
  If the willingness to get a vaccine is there, in principle, but the problem is one of getting organized, we can use a simplified version of the good old problem-solving scheme. Starting out by repeating the aim (i.e. to “get a vaccine as soon as possible”) and defining the barriers (“haven’t found the time yet” or “can’t find vaccine booklet” etc.), continue with a brainstorming of different options of where, when and how the aim can be achieved, motivate the person to think about the disadvantages and advantages of each option, then encourage to select an option and outline a concrete plan that includes the specific steps involved along with a time and place. If possible, support the person practically (e.g. find the closest doctor offering vaccines) and don’t forget to follow up by asking whether the plan was put into practice. If not, elucidate the reasons, select a new option and take it from there.

Does this sound straightforward enough to give it a go? If so, we are curious to hear whether it worked and are awaiting your “case-reports” in the next issue.

## References

[r1] Allington, D., Duffy, B., Wessely, S., Dhavan, N., & Rubin, J. (2021). Health-protective behaviour, social media usage and conspiracy belief during the COVID-19 public health emergency. Psychological Medicine, 51(10), 1763–1769. 10.1017/S003329172000224X 32513320 PMC7298098

[r2] Bono, S. A., Faria de Moura Villela, E., Siau, C. S., Chen, W. S., Pengpid, S., Hasan, M. T., Sessou, P., Ditekemena, J. D., Amodan, B. O., Hosseinipour, M. C., Dolo, H., Siewe Fodjo, J. N., Low, W. Y., & Colebunders, R. (2021). Factors affecting COVID-19 vaccine acceptance: An international survey among low- and middle-income countries. Vaccines, 9(5), 515. 10.3390/vaccines9050515 34067682 PMC8157062

[r3] COSMO-Konsortium. (2021, September 20). *Zusammenfassung und Empfehlungen Wellen 48 bis 51*. https://projekte.uni-erfurt.de/cosmo2020/web/summary/48-51/

[r4] Ebrahimi, O. V., Johnson, M. S., Ebling, S., Amundsen, O. M., Halsøy, Ø., Hoffart, A., Skjerdingstad, N., & Johnson, S. U. (2021). Risk, trust, and flawed assumptions: Vaccine hesitancy during the COVID-19 Pandemic. Frontiers in Public Health, 9, 700213. 10.3389/fpubh.2021.700213 34277557 PMC8281037

[r5] Freeman, D., Loe, B. S., Chadwick, A., Vaccari, C., Waite, F., Rosebrock, L., Jenner, L., Petit, A., Lewandowsky, S., Vanderslott, S., Innocenti, S., Larkin, M., Giubilini, A., Yu, L.-M., McShane, H., Pollard, A. J., & Lambe, S. (2020). COVID-19 vaccine hesitancy in the UK: The Oxford coronavirus explanations, attitudes, and narratives survey (Oceans) II. Psychological Medicine. Advance online publication. 10.1017/S0033291720005188 33305716 PMC7804077

[r6] Holzmann-Littig, C., Braunisch, M. C., Kranke, P., Popp, M., Seeber, C., Fichtner, F., Littig, B., Carbajo-Lozoya, J., Allwang, C., Frank, T., Meerpohl, J. J., Haller, B., & Schmaderer, C. (2021). COVID-19 vaccination acceptance and hesitancy among healthcare workers in Germany. Vaccines, 9(7), 777. 10.3390/vaccines9070777 34358193 PMC8310090

[r7] Lincoln, T. M., Schlier, B., Strakeljahn, F., Gaudiano, B., So, S., Kingston, J., Morris, E., & Ellett, L. (2021). A machine learning approach to identify psychological factors driving vaccine hesitancy in high income countries. Research Square. 10.21203/rs.3.rs-787170/v1 PMC8827083 35136120

[r8] Murphy, J., Vallières, F., Bentall, R. P., Shevlin, M., McBride, O., Hartman, T. K., McKay, R., Bennett, K., Mason, L., Gibson-Miller, J., Levita, L., Martinez, A. P., Stocks, T. V. A., Karatzias, T., & Hyland, P. (2021). Psychological characteristics associated with COVID-19 vaccine hesitancy and resistance in Ireland and the United Kingdom. Nature Communications, 12(1), 29. 10.1038/s41467-020-20226-9 33397962 PMC7782692

[r9] Neumann-Böhme, S., Varghese, N. E., Sabat, I., Barros, P. P., Brouwer, W., van Exel, J., Schreyögg, J., & Stargardt, T. (2020). Once we have it, will we use it? A European survey on willingness to be vaccinated against COVID-19. The European Journal of Health Economics, 21(7), 977–982. 10.1007/s10198-020-01208-6 32591957 PMC7317261

[r10] Peretti-Watel, P., Seror, V., Cortaredona, S., Launay, O., Raude, J., Verger, P., Fressard, L., Beck, F., Legleye, S., L’Haridon, O., Léger, D., & Ward, J. K. (2020). A future vaccination campaign against COVID-19 at risk of vaccine hesitancy and politicisation. The Lancet Infectious Diseases, 20(7), 769–770. 10.1016/S1473-3099(20)30426-6 32445713 PMC7239623

[r11] Prochaska, J. O., & Diclemente, C. C. (1986). Toward a comprehensive model of change. In W. R. Miller & N. Heather (Eds.), *Treating addictive behaviors: Processes of change* (pp. 3–27). Springer US. 10.1007/978-1-4613-2191-0_1

[r12] Rief, W. (2021). Fear of adverse effects and COVID-19 vaccine hesitancy: Recommendations of the treatment expectation expert group. JAMA Health Forum, 2(4), e210804. 10.1001/jamahealthforum.2021.0804 36218819

[r13] Rief, W., & Joormann, J. (2019). Revisiting the cognitive model of depression: The role of expectations. Clinical Psychology in Europe, 1(1), e32605. 10.32872/cpe.v1i1.32605

[r14] Sallam, M. (2021). COVID-19 vaccine hesitancy worldwide: A concise systematic review of vaccine acceptance rates. Vaccines, 9(2), 160. 10.3390/vaccines9020160 33669441 PMC7920465

[r15] Wake, A. D. (2021). The willingness to receive COVID-19 vaccine and its associated factors: “Vaccination refusal could prolong the war of this pandemic” – A systematic review. Risk Management and Healthcare Policy, 14, 2609–2623. 10.2147/RMHP.S311074 34188572 PMC8232962

